# Integrative bioinformatics and *in vitro* exploration of EVI2A expression: unraveling its immunological and prognostic implications in kidney renal clear cell carcinoma

**DOI:** 10.32604/or.2024.050851

**Published:** 2024-10-16

**Authors:** RONG LIU, SHENG LI, SITU XIONG, FUCUN ZHENG, XIANGPENG ZHAN, JIN ZENG, BIN FU, SONGHUI XU, SHAOXING ZHU, RU CHEN

**Affiliations:** 1 Department of Urology, Fujian Medical University Union Hospital, Fuzhou, 350000, China; 2 Department of Urology, The First Affiliated Hospital of Nanchang University, Nanchang, 350000, China

**Keywords:** EVI2A, Kidney Renal Clear Cell Carcinoma (KIRC), Prognosis, Immunological analysis

## Abstract

EVI2A has emerged as a significant biomarker in various diseases; however, its biological role and mechanism in kidney renal clear cell carcinoma (KIRC) remains unexplored. We used TCGA and GEO databases to analyze EVI2A gene expression comprehensively and performed pan-cancer assessments. Clinical relevance was evaluated through Kaplan-Meier analysis and ROC curves. The gene’s immune relevance was explored through analyses of the tumor microenvironment (TME), Tumor Immune Single-cell Hub (TISCH), immune checkpoints, and immunotherapy sensitivity. Our results indicate that EVI2A expression is upregulated in KIRC, showing correlations with tumor grade and T/N/M stage. EVI2A demonstrates high diagnostic accuracy (AUC=0.906) and predicts poor overall and progression-free survival in KIRC patients. Furthermore, EVI2A expression exhibits significant associations with immunity, including TME scores and specific immune cell types such as Tfh cells, CD4 memory T cells, and CD8+ T cells. Elevated EVI2A expression suggests increased sensitivity to PD-1/CTLA-4 and tyrosine kinase inhibitors. *In vitro* assays confirmed the impact of EVI2A on KIRC behavior, with its knockdown resulting in reduced cell proliferation and migration. In conclusion, our comprehensive analysis identifies EVI2A as a promising biomarker and a novel therapeutic target for intervening in KIRC. These findings hold significant implications for further research and potential clinical applications.

## Introduction

Renal cell carcinoma, the predominant and lethal urologic malignancy [1], constitutes approximately five percent of global cancer cases [2]. In recent decades, there has been a continuous escalation in the incidence of kidney cancer. In 2020 alone, around 431,000 new cases were diagnosed globally, with an annual occurrence of approximately 175,000 kidney cancer-related deaths [3]. Projections indicate a further surge in the incidence and mortality of RCC, estimating over 300,000 deaths by 2040 [3,4].

The predominant pathological manifestation of renal cell carcinoma is kidney renal clear cell carcinoma (KIRC), encompassing over 70% of all cases [5]. While localized renal carcinoma can be effectively managed through surgical resection, metastatic forms exhibit resistance to conventional radiotherapy and chemotherapy [6]. Alarmingly, up to 20%–30% of patients present with distant metastases at the time of diagnosis. Moreover, 30 percent of individuals with localized renal cancer, despite surgical intervention, confront postoperative recurrence and progression to metastatic renal cancer [7].

Although immunotherapy and targeted therapies offer a beacon of hope for kidney cancer patients, promising enhanced efficacy and safety compared to traditional treatments, not all patients experience durable remission rates [8]. Therefore, it is crucial to keep searching for new prognostic and other possible therapeutic biomarkers. Research findings have illuminated the overexpression of Ecotropic Viral Integration Site 2A (EVI2A) in certain neoplasms, positioning it as a promising prognostic marker. Pasmant et al. [9] have posited that EVI2A might elevate the susceptibility to peripheral nerve sheath malignancies in individuals with nf1 deficiency, though its precise function remains elusive. Zhou et al. [10] have advanced the notion of EVI2A as an environmentally relevant gene influencing the prognosis of esophageal cancer. Additionally, existing studies [11,12] suggest EVI2A’s role as a methylation-related gene linked to the prognosis of KIRC, yet a more nuanced analysis is requisite.

**Figure 1:**
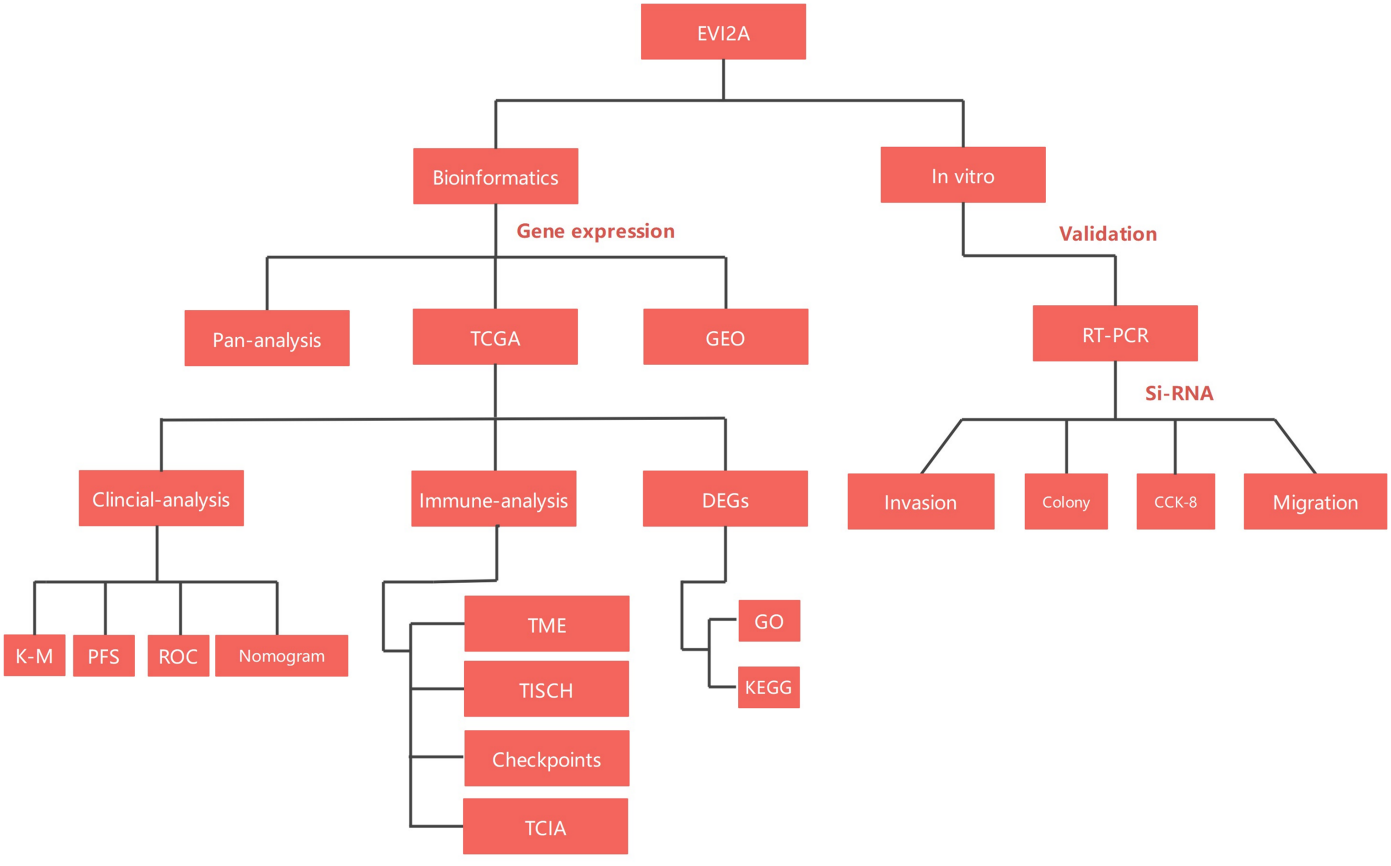
Flow-process diagram of the study.

In our investigation, we conducted an exhaustive bioinformatics analysis of EVI2A, followed by validating its biological implications in KIRC through cellular functional experiments. This study will contribute to a more profound comprehension of the potential role of EVI2A in tumor pathogenesis. Furthermore, we aspire to enhance the molecular targeting therapy and prognosis for KIRC patients. The flowchart of the study is illustrated in Fig. 1.

## Materials and Methods

### Analysis of EVI2A expression in renal clear cell carcinoma and pan carcinoma

The mRNA expression of EVI2A in various cancers, including KIRC, was obtained from TIMER 2.0 (http://timer.comp-genomics.org/) database. Besides, the gene expression files and associated clinical data of KIRC patients were downloaded from the TCGA database and GEO database (GSE66272, GSE53757, GSE53000, GSE68417). A significant finding was defined as a *p*-value below 0.05.

### Analysis of clinical associations

In the initial phase, we employed the median value of EVI2A expression as the threshold to dichotomize patients into cohorts exhibiting high and low expression levels. Subsequently, the Kaplan-Meier (K-M) method was utilized to elucidate the influence of EVI2A expression on progression-free survival (PFS) and overall survival (OS). Following this, we analyzed the correlation between EVI2A and clinicopathological features. In the concluding stage, the clinical utility of the gene was verified by diagnostic ROC. All analyses were performed using R version 4.1.3, and the corresponding R code and raw data are accessible for download in the website (https://www.jianguoyun.com/p/Daw2ZlQQ0tqcDBjwh6sFIAA).

### Functional enrichment analysis and co-expression network construction

We conducted a rigorous differential analysis to discern the distinctive gene expression profiles between cohorts characterized by high and low levels of EVI2A expression. Subsequently, we delved into the functional characterization of these differentially expressed genes (DEGs) through comprehensive Gene Ontology (GO) and Kyoto Encyclopedia of Genes and Genomes (KEGG) analyses. These analyses aimed to unravel the intricate biological mechanisms associated with EVI2A. To identify genes co-expressed with EVI2A, we calculated Spearman correlation coefficients, selecting those with a magnitude greater than 0.75 (|R2 | > 0.75) and a significance level below 0.001 (*p* < 0.001). The intricate relationship between EVI2A and its co-expressed genes was elegantly visualized using the “circlize” R package.

### Exploring the interplay between EVI2A expression and the tumor microenvironment alongside the immune system

Tumor microenvironment (TME) scores were meticulously computed utilizing the “estimate” R package, and distinctions in TME characteristics between groups with high and low EVI2A expression were discerned through the “limma” R package. The Tumor Immune Single Cell Center (TISCH), a scRNA-seq database dedicated to scrutinizing the Tumor Microenvironment (TME) [13], played a pivotal role in this investigation. By delivering comprehensive annotations of cell types at the single-cell level, TISCH facilitated a nuanced exploration of TME intricacies across diverse cancer types. This database was instrumental in further scrutinizing TME heterogeneity concerning various treatments, primary and metastatic sites at the single-cell level, and investigating the interplay between EVI2A and immune cells. Additionally, we delved into the correlation between EVI2A and immune checkpoints, leveraging TCIA data [14,15] to prognosticate the relationship between EVI2A expression and immunotherapy sensitivity.

### Assessing drug sensitivity

To appraise drug sensitivity, we employed the “pRRophetic” package within the R software to predict the half-maximal inhibitory concentration (IC50) values for prevalent chemotherapy drugs and tyrosine kinase inhibitors (TKIs) across both high- and low-risk groups. Significance was attributed to *p* values below 0.05, and these statistically meaningful distinctions were visually represented by creating box plots.

### Cell culture and cell transfection

The human renal cancer cell lines 786-0 (#CL-0010), 0SRC-2 (#CL-0177), CAKI-1 (#CL-0052), ACHN (#CL-0021), and HK-2 cells (#CL-0109) were purchased from Procell Life Science & Technology (Wuhan, China). We conducted mycoplasma testing and regularly tested all cell lines during the experiments to ensure a contamination-free culture environment. Additionally, we authenticated the cell lines using STR (short tandem repeat) analysis to confirm their origin and accuracy. 786-0 and OSRC-2 cells were cultured in RPMI-1640 (#C11875500BT, Thermo Fisher Scientific, Waltham, USA) supplemented with 10% fetal bovine serum (FBS, # 16000-044, Invitrogen, USA). ACHN cells were cultured in MEM (#11095080, Thermo Fisher Scientific, Waltham, USA) supplemented with 10% FBS. CAKI-1 and HK-2 cells were cultured in DMEM (#11965092, Thermo Fisher Scientific, Waltham, USA) supplemented with 10% FBS. All cells were cultured in a humidified incubator at 37°C with 5% CO_2_. Targeted small interfering RNA (siRNA) against EVI2A was transiently transfected into cells using Lipofectamine 2000 (Invitrogen, USA). The sequences of siRNA and EVI2A are shown in Table 1.

**Table 1:** EVI2A and Si-EVI2A sequences

Genes	Sequence	
EVI2A	GCGATTTTCTGGCAAGCGGTCT	TCCCTTGTAGCTGTGAGCACTC
S1-EVI2A	5′CCCTATAACTCCTGAAGTA′3	
S2-EVI2A	5′GACCCAACCTAGTGATGCA′3	

### RNA isolation and quantitative real-time PCR (RT-qPCR)

The study involved obtaining 10 pairs of adjacent normal and cancerous tissue specimens from the First Affiliated Hospital of Nanchang University in China. Ethical approval for the collection and uses of all biological samples in this research was granted by the Ethics Committee of the First Affiliated Hospital of Nanchang University (Approval: (2023) CDYFYYLK (03-013)). The procedures were conducted in accordance with the informed consent of the participants. Total RNA was extracted using Invitrogen TRIzol (#15596026, Thermo Fisher Scientific, Waltham, USA) reagent and cDNA synthesis was performed using the Takara PrimeScript RT kit (#RR037A, Beijing, China). RT-qPCR was performed using SYBR Green (Roche, Switzerland). The relative expression of EVI2A gene was calculated using the 2^−ΔΔCt^ method with ACTIN as the reference gene.

### Western blotting

RIPA extraction reagent (Beyotime, Shanghai, China) was used to lyse cells and extract total protein. The protein concentration was determined using the BCA Protein Assay (TIANGEN, Beijing, China). Subsequently, samples underwent SDS-PAGE, and the proteins were transferred onto a polyvinylidene difluoride (PVDF) membrane (Cytiva, UK), followed by blocking with 5% BSA for 2 h. The membranes, along with primary antibodies (anti-EVI2A: 1:800, #17415-1-AP, and anti-β-actin: 1:3000, #20536-1-AP, ProteinTech, Wuhan, China), were incubated overnight at 4°C. Afterward, the membranes were incubated with the secondary antibody (goat anti-rabbit IgG-HRP, 1:3000, Cell Signaling Technology, Boston, USA) for 1.5 h at room temperature. The process was concluded by manual exposure in a darkroom.

### Cell counting kit-8 (CCK8) assay and colony formation assay

We followed the recommended protocol and assessed cell proliferation using the CCK-8 kit (#K1018, Apexbio, USA). 786-O and ACHN cells were seeded at a density of 1000 cells/well in 96-well plates and cell viability was measured on days 0, 1, 2, 3 and 4. To evaluate the colony formation ability, 1000 cells were seeded in 6-well plates and treated differently for two weeks. The colonies were fixed using 4% paraformaldehyde (#P0099, Beyotime, Shanghai, China), stained with 0.1% crystal violet (#C0121, Beyotime, Shanghai, China), and quantified using ImageJ software.

### Cell migration assay

In the transwell experiment, a cell suspension comprising 40,000 cells was supplemented with an FBS-free medium to a volume of 200 µL. This mixture was evenly inoculated into the upper chamber, while the lower chamber received 800 µL of medium containing 20% FBS. Following a 24-h incubation period, the cells were fixed with methanol and stained using 0.1% crystal violet. The lower surface of the chamber was photographed, and cell migration was assessed through meticulous counting under a microscope. Post-transfection, cells were cultured in 6-well plates until reaching 90% confluence. A 1 ml pipette tip was employed to create a scratch, and any debris was subsequently removed with PBS buffer. Following this, 1 mL of serum-free medium was added to each well. Photographic documentation occurred at 0- and 24-h post-injury, and the wound area was precisely calculated utilizing ImageJ software.

### Statistical analysis

All bioinformatics analyses were performed through R language software(Version 4.1.3), except for the previously mentioned online analysis site. The students’ *t*-tests were used to compare each group *in vitro* experiments, visualized through GraphPad Prism software (Version 8.0). Statistical significance was set at a *p*-value < 0.05.

## Results

### Elevated expression of EVI2A in kidney renal clear cell carcinoma (KIRC)

**Figure 2:**
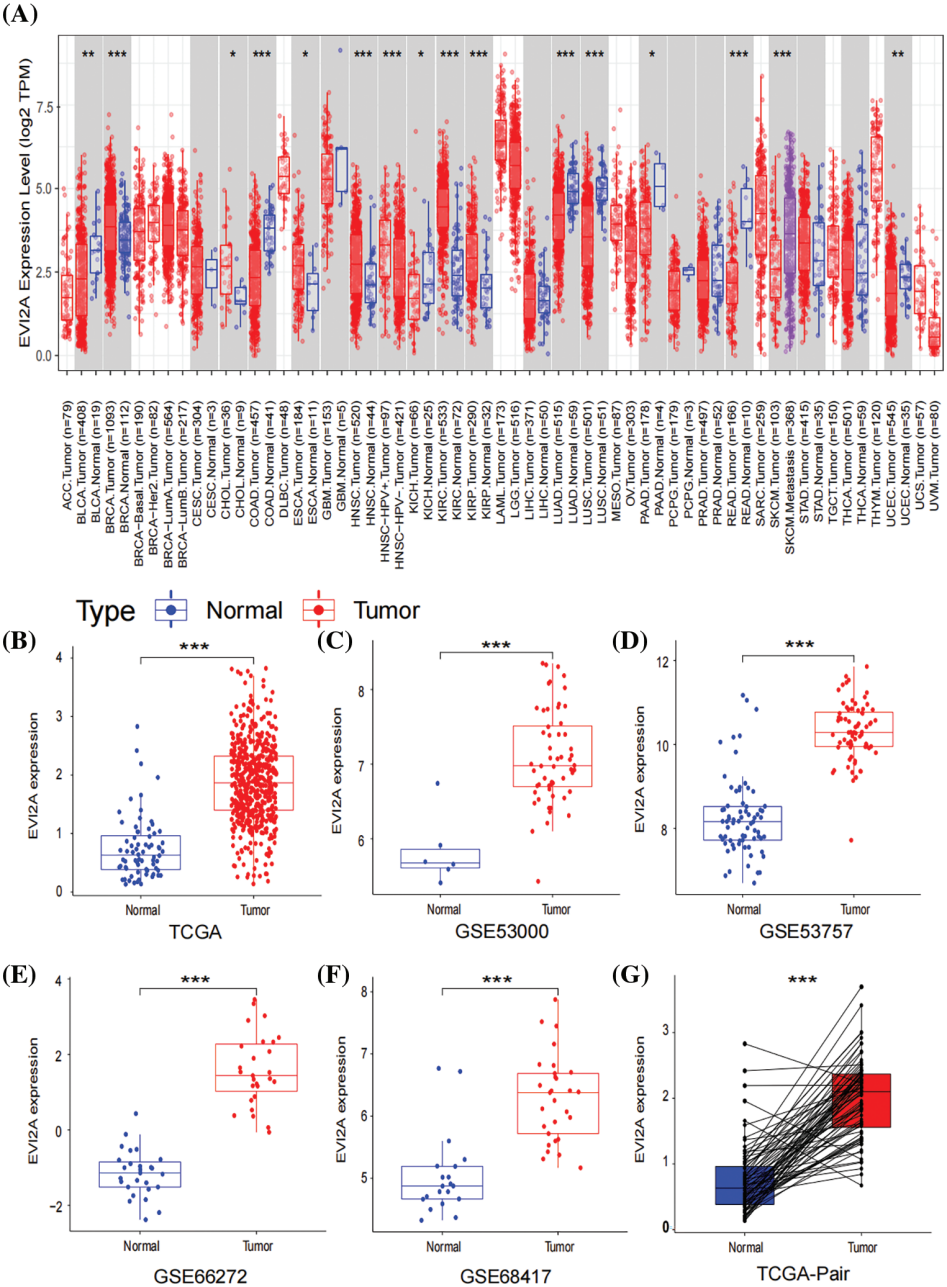
Expression of EVI2A in cancers. (A) Expression levels of EVI2A in different tumors and normal tissues. (B, G) The differential expression of EVI2A from the TCGA database and (C–F) GEO database. *p < 0.05, **p < 0.01, ***p < 0.001.

As illustrated in Fig. 2A, our pan-cancer analysis unveiled a conspicuous upregulation of EVI2A expression not only in KIRC but also in other malignancies such as breast cancer (BRCA), Head and Neck Squamous Cell Carcinoma (HNSC), Cholangiocarcinoma (CHOL), and Kidney Renal Papillary Cell Carcinoma (KIRP). To delve deeper into the expression profile of EVI2A specifically in KIRC, we curated a cohort comprising 922 KIRC patients from TCGA (comprising 72 normal tissue samples and 541 tumor tissue samples) and GEO datasets (including GSE 53000 with six normal tissue samples and 56 tumor tissue samples, GSE5375 with 72 normal tissue samples and 72 tumor tissue samples, GSE66272 with 27 normal tissue samples and 27 tumor tissue samples, and GSE68417 with 20 normal tissue samples and 29 tumor tissue samples). The detailed dataset information is presented in Suppl. Table S1.

As depicted in Figs. 2B–2F, a statistically significant elevation of EVI2A expression in KIRC was observed when comparing tumor and normal tissue samples, a trend further substantiated by the results from paired samples (Fig. 2G).

### High EVI2A expression is associated with poor outcomes in KIRC patients

**Figure 3:**
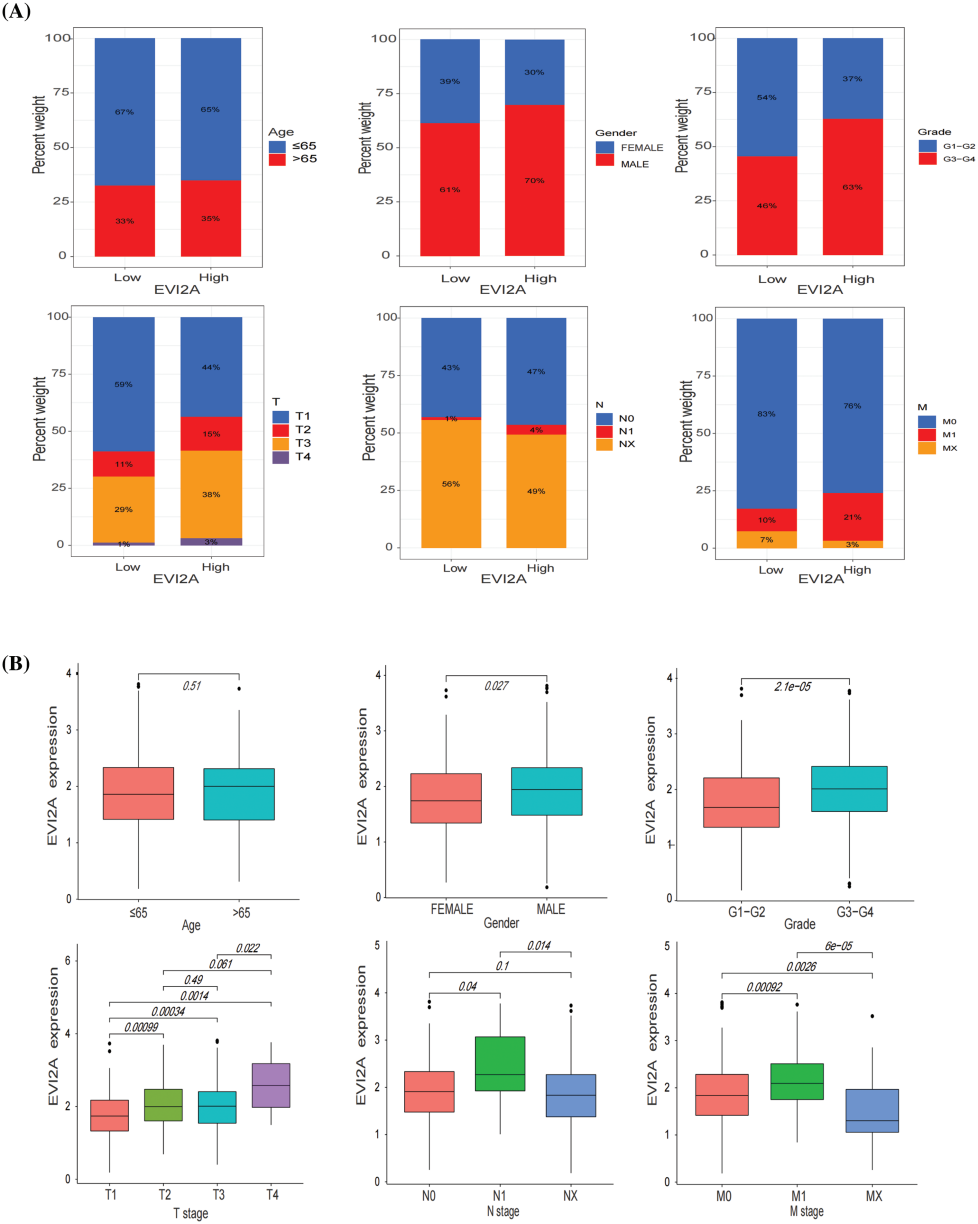
Relationship between EVI2A expression and clinicopathological features including age, gender, grade, and T/N/M stage (A and B).

To elucidate the association between EVI2A expression and clinical baseline parameters (age, gender) as well as pathological features (T/N/M stage, grade), we employed bar and box plots for visual representation. As illustrated in Fig. 3A, the proportion of the M1 stage in the high EVI2A expression group was twofold higher than that in the low expression group (21% *vs*. 10%). Similarly, the prevalence of advanced T stage and high pathological grade in the high-level group surpassed that in the low-level group (56% *vs*. 41%; 63% *vs*. 46%). Conversely, the percentages of age, gender, and N stage exhibited no significant differences between the two groups. Fig. 3B illustrates a significant association between elevated EVI2A expression in KIRC and TNM stage and tumor grade (*p* < 0.05). This underscores a positive correlation between increased EVI2A expression and advanced TNM stage, excluding cases with unknown TNM staging and pathological grading.

**Figure 4:**
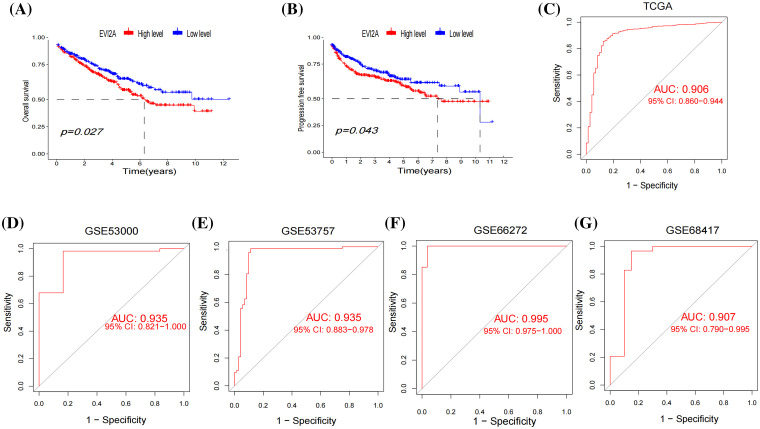
Clinical analysis of EVI2A. (A) The Kaplan–Meier curves about the correlation between EVI2A expression and overall survival and (B) progression-free survival. (C–G) The diagnosis ROC of EVI2A in TCGA and GEO databases.

Leveraging the comprehensive clinical information and large sample size (n = 613) of the TCGA dataset, we investigated the correlation between EVI2A expression and the prognosis of KIRC patients. Kaplan–Meier analysis unveiled a marked association between high EVI2A expression and poorer overall survival (OS) (*p* < 0.05; Fig. 4A) and progression-free survival (PFS) (*p* < 0.05; Fig. 4B). Univariate and multivariate Cox regression analyses explored whether EVI2A expression and clinicopathological features were independent factors in KIRC patients (Suppl. Fig. S1). In univariate Cox analysis, EVI2A expression level, pathological grade, T/M stage, and age emerged as independent prognostic factors. The multiple Cox regression analysis identified factors other than EVI2A as independent prognostic indicators influencing KIRC. Moreover, diagnostic AUCs of EVI2A exceeded 0.9 in both TCGA and GEO databases, as illustrated in Figs. 4C–4G.

### KEGG, GO and co-expressed analysis of EVI2A-related genes

**Figure 5:**
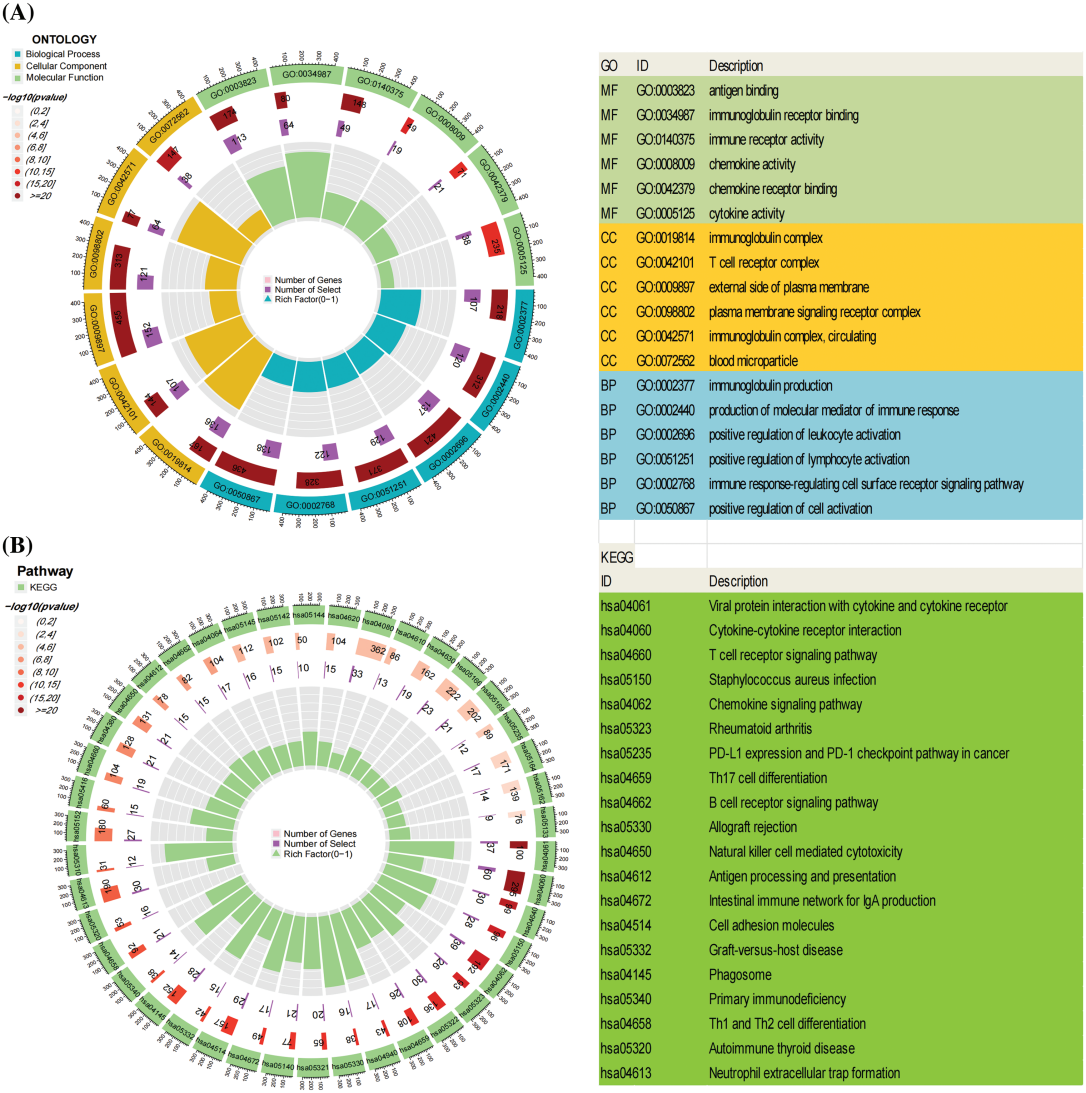
The Gene Ontology (GO) and Kyoto Encyclopedia of Genes and Genomes (KEGG) enrichment analysis of Differentially Expressed Genes (DEGs). (A) illustrates the top six enriched pathways in Molecular Function (MF), Cellular Component (CC), and Biological Process (BP). (B) presents the top 20 pathways in the KEGG enrichment analysis.

Following the screening process, a total of 1669 Differentially Expressed Genes (DEGs) were identified (| LogFC | > 2, *p* < 0.05, refer to Suppl. Table S2 for details). The Gene Ontology (GO) enrichment analysis encompasses an exploration of the molecular function (MF), cellular component (CC), and biological process (BP) associated with the identified genes. Illustrated in the circular diagram (Fig. 5A) are the six processes exhibiting the most significant enrichment among these three components. GO enrichment analysis reveals a close correlation between the more enriched DEGs and immune processes, including immunoglobulin receptor binding, immune receptor activity, immune response-regulating cell surface receptor signaling pathway, and others. Additionally, Kyoto Encyclopedia of Genes and Genomes (KEGG) enrichment analysis underscores the significant involvement of DEGs in immune-related pathways such as Th1 and Th2 cell differentiation, T cell receptor signaling pathway, PD-L1 expression, PD-1 checkpoint pathway in cancer, and B cell receptor signaling pathway (Fig. 5B). Detailed raw data are available in Suppl. Tables S3–S4.

These outcomes strongly suggest that the identified DEGs are intricately linked to immune-related biological processes, providing robust support for the notable correlation between EVI2A and immune responses. In the co-expression analysis of genes, we isolated the top five genes positively and negatively associated with EVI2A, presenting them visually in circular plots (Suppl. Fig. S2, Table S5). TNFSF13B, MS4A6A, CD86, RGS18, and GPR65 exhibited positive associations with EVI2A, whereas TMEM8B, HDAC11, GNA11, CKB, and RAP1GAP displayed negative associations with EVI2A.

### Relationship between EVI2A expression and heterogeneity of tumor immune microenvironment

**Figure 6:**
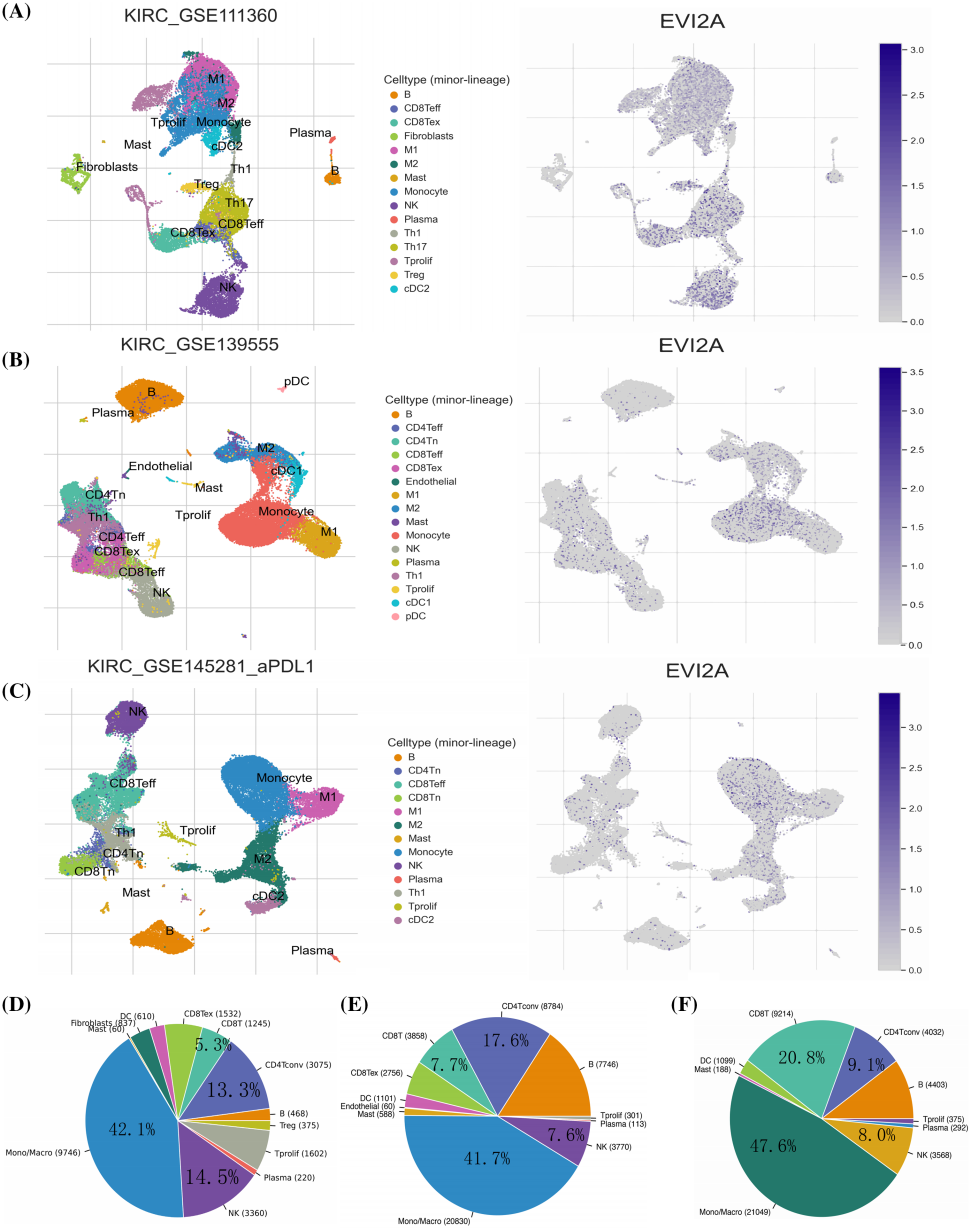
Research results of EVI2A at single-cell level. Single-cell cluster map and percentage chart of EVI2A in (A, D) KIRC_GSE111360, (B, E) KIRC_GSE139555, and (C, F) KIRC_GSE145281_aPDL1.

Initially, we leveraged three datasets (KIRC_GSE111360, KIRC_GSE139555, and KIRC_GSE145281_aPDL1) from the TISCH database to evaluate EVI2A expression within Tumor Microenvironment (TME)-associated immune cells. As depicted in Figs. 6A–6C, EVI2A manifested predominant expression in CD8+ T cells, CD4+ T cells, and macrophages. Moreover, we observed varying but consistently detectable levels of EVI2A expression in other immune cells, encompassing NK, B, and Mast cells.

Quantitative analysis of EVI2A expression across the various cell groups is presented in Suppl. Fig. S3. The distribution and abundance of diverse cells associated with the TME are illustrated in Figs. 6D–6F. Notably, CD8T cells exhibited significantly higher prevalence in GSE145281 than in GSE111360 and GSE139555 (20.8% *vs*. 7.7% *vs*. 5.3%). This discrepancy may be attributed to the fact that GSE145281 [16] primarily includes patients with metastatic kidney cancer undergoing immunotherapy. At the same time, GSE111360 [17] and GSE139555 [18] primarily consist of individuals with primary kidney cancer who did not receive immunotherapy. This observation highlights distinct TME-related cell profiles in primary and metastatic KIRC.

**Figure 7:**
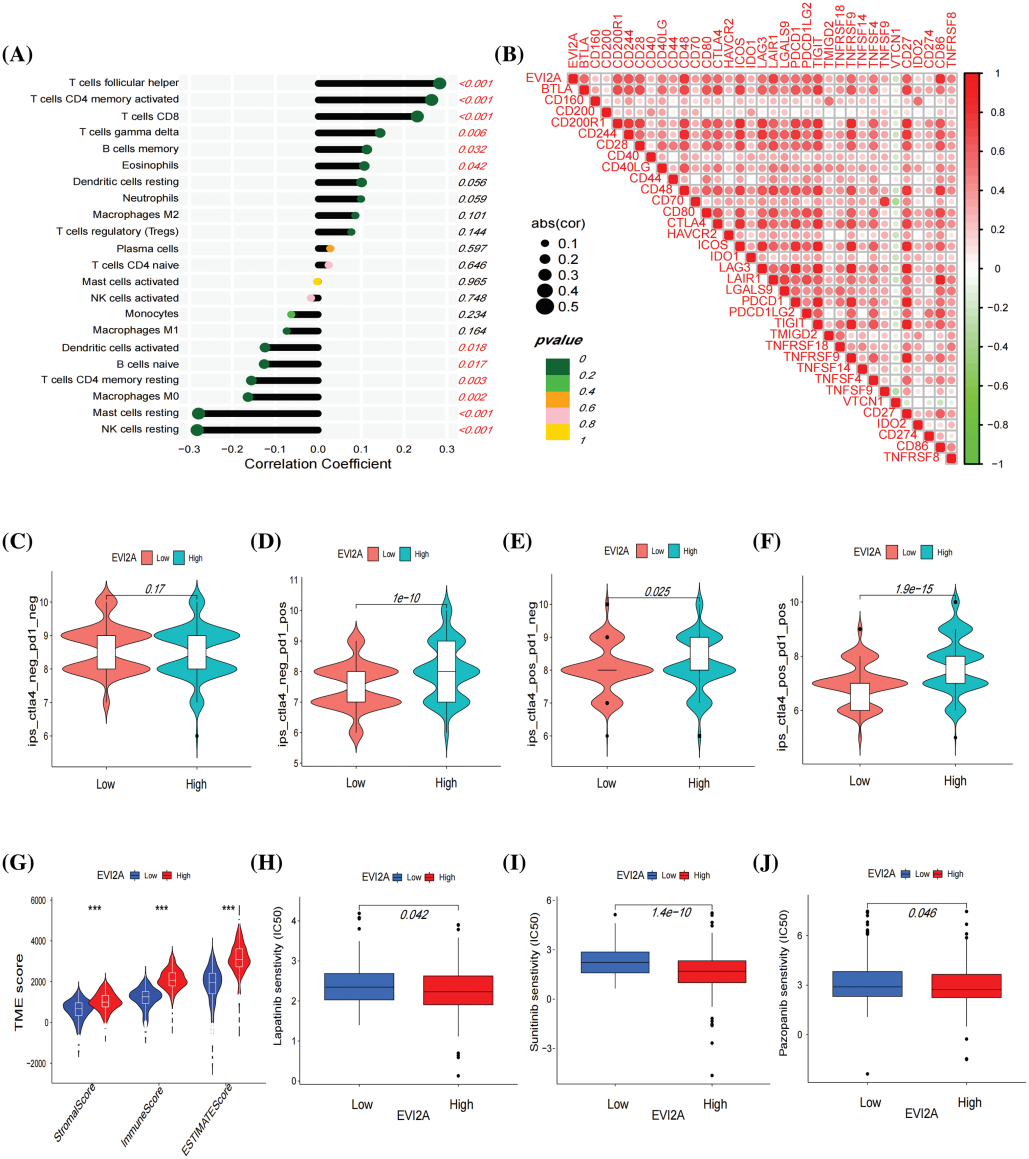
Immune-related analysis of EVI2A. (A) Correlation between EVI2A expression and immune cells. (B) Correlation between EVI2A and the most common immune checkpoints. (C–F) Immunotherapy sensitivity analysis based on TCIA database in high and low expression EVI2A groups. (G) Analysis of tumor microenvironment in high and low expression EVI2A groups. (H–J) Drug sensitive analysis. ***p < 0.001.

Subsequently, Spearman correlation analysis was employed to scrutinize the correlation between EVI2A expression and immune cells, with the results presented in Suppl. Table S6 and Fig. 7A. The outcomes revealed a positive correlation between EVI2A expression and various immune cell subtypes, including T cells follicular helper (Tfh), CD4 memory activated T cells, CD8 T cells, T cells gamma delta, B cells memory, and Eosinophils. Conversely, NK cells resting, Macrophages M0, Mast cells resting, T cells CD4 memory resting, and B cells naive negatively correlated with EVI2A expression. Additionally, results computed using the ESTIMATE algorithm indicated higher stromal scores, immune scores, and ESTIMATE-Scores in the high expression EVI2A group (Fig. 7G).

### EVI2A correlates with immune checkpoints, immunotherapy, and targeted therapy sensitivity in kidney renal clear cell carcinoma (KIRC)

To scrutinize the relationship between EVI2A and immunotherapy, we conducted a comprehensive analysis examining the correlation between EVI2A and prevalent immune checkpoints. Notably, EVI2A exhibited positive associations with a diverse array of immune checkpoints, including but not limited to CTLA4, PDCD1, PDCD1LG2, LAG3, CD200R1, and TNFRSF4. Notably, VTCN1 was the sole exception, displaying a negative association with EVI2A (Fig. 7B; refer to Suppl. Table S7 for details). The Cancer Immunome Atlas (TCIA) provided extensive immunogenomic analysis results from next-generation sequencing data encompassing multiple solid tumor types and 9562 cases from TCGA and other sources. When at least one of CTLA-4 or PD-1 is positive, high expression of EVI2A is associated with increased sensitivity to immunotherapy (Figs. 7C–7F). Additionally, the findings from drug sensitivity analysis indicated that individuals with high EVI2A expression presented lower half-maximal inhibitory concentration (IC50) values for drugs such as sunitinib, pazopanib, and lapatinib in comparison to those with low expression (Figs. 7H–7J).

### Inhibition of EVI2A reduces the viability and migration in renal cancer cells

**Figure 8:**
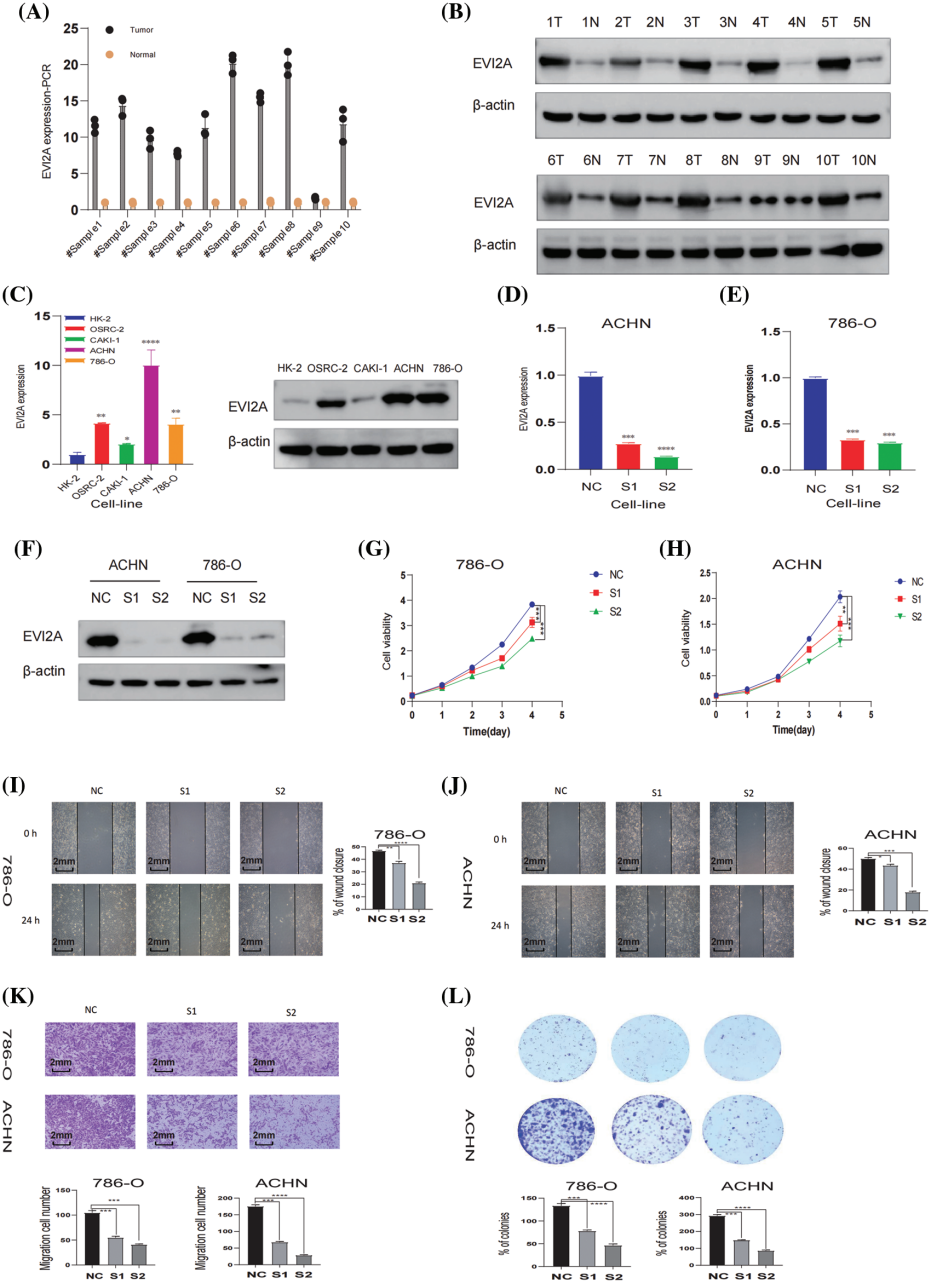
*In vitro* analysis of EVI2A. (A) Quantitative real-time polymerase chain reaction (qRT-PCR) analysis depicting EVI2A expression in normal kidney tissue and kidney cancer tissue. (B) Western Blot (WB) analysis illustrating EVI2A protein levels in normal kidney tissue and kidney cancer tissue. (C) qRT-PCR and WB analysis demonstrating EVI2A expression in normal kidney cells and kidney cancer cell lines. (D–F) Verification of relative EVI2A expression through qPCR and WB in 786-O and ACHN cell lines post-transfection with S1-EVI2A and S2-EVI2A. (G and H) Growth curves of 786-O and ACHN cell lines assessed via Cell Counting Kit-8 (CCK-8) following transfection with designated vectors. (I–K) Evaluation of cell migration ability using wound healing and transwell assays. (L) Colony formation assay determining cell proliferation ability post-transfection with specified vectors. *p < 0.05; **p < 0.01; ***p < 0.001; ****p < 0.0001.

As illustrated in Figs. 8A and 8B, the Western blotting (WB) and RT-qPCR results from 10 pairs of renal cancer and adjacent tissues indicate elevated EVI2A expression in renal cancer tissues. Furthermore, we compared relative mRNA levels between normal kidney cells (HK-2) and various renal cancer cell lines, including CAKI-1, OS-RC-2, 786-O, and ACHN, as shown in Fig. 8C. These findings highlight a pronounced increase in EVI2A levels in tumor cells compared to HK-2. Three small interfering RNAs (siRNAs), namely s1-EVI2A, s2-EVI2A, and s3-EVI2A, were meticulously designed to target the junction sites of EVI2A specifically. The quantitative Reverse Transcription Polymerase Chain Reaction (qRT-PCR) results demonstrated that both s1 and s2 significantly reduced the expression level of EVI2A (Figs. 8B–8C). Consequently, s1 and s2 were selected for subsequent cell function experiments. The inhibitory effect on EVI2A led to a noteworthy reduction in migration ability, as evidenced by the results presented in Figs. 8E–8F. Furthermore, assessments using Cell Counting Kit-8 (CCK-8) and colony formation assays revealed that the proliferation of ACHN and 786-O cell lines was distinctly attenuated following the knockdown of EVI2A (Figs. 8D, 8G). In summary, these findings unequivocally underscore the indispensable role of EVI2A in governing the viability, migration, and survival of kidney cancer cells.

## Discussion

Despite considerable progress in surgical planning and comprehensive treatment modalities for kidney cancer, these alternatives still fall short of being optimal for locally advanced and metastatic kidney cancer, posing a formidable challenge for urologists. Research indicates that patients with metastatic renal cell carcinoma exhibit a 5-year overall survival rate of less than 20%, with a median overall survival not exceeding 1.5 years [19,20]. Consequently, there is an imperative need to explore efficacious biomarkers for kidney cancer and propose more diverse anti-tumor therapeutic options. Our investigation has pinpointed EVI2A as a novel molecular marker for renal clear cell carcinoma, identified through meticulous analysis of the TCGA database. Furthermore, we substantiated that EVI2A exerts a discernible impact on renal cancer cell function through meticulously designed *in vitro* experiments. Analysis of data from the GEO and TCGA databases revealed an upregulation of EVI2A expression in renal clear cell carcinoma tissues compared to adjacent normal tissues. Additionally, *in vitro* experiments consistently validated the heightened expression of EVI2A in renal clear cell carcinoma cells compared to normal kidney cells.

Through clinical correlation analysis, our initial observation revealed a substantial correlation between the upregulation of EVI2A mRNA and advanced T/N/M-stage and a high-grade pathological stage in Kidney Renal Clear Cell Carcinoma (KIRC). Subsequently, both the survival and progression-free survival curves indicated that heightened EVI2A expression was closely associated with a less favorable prognosis (*p* < 0.05). The consistency between these two outcomes reinforces the robustness of our findings. The diagnostic Receiver Operating Characteristic (ROC) analysis of EVI2A yielded significant results in both The Cancer Genome Atlas (TCGA) and externally validated Gene Expression Omnibus (GEO) datasets, suggesting that EVI2A could serve as a novel diagnostic biomolecule for KIRC. To delve deeper into the biological functions of EVI2A, we conducted Gene Ontology (GO) and Kyoto Encyclopedia of Genes and Genomes (KEGG) enrichment analyses. The outcomes underscored the close association of EVI2A with various immune-related pathways, including immunoglobulin receptor binding, PD-L1 expression, PD-1 checkpoint pathway in cancer, and the T cell receptor signaling pathway.

As commonly acknowledged, renal cell carcinoma exhibits limited responsiveness to chemotherapy and radiotherapy [21]. Immunotherapeutic agents have demonstrated robust efficacy, yielding superior treatment outcomes in metastatic renal cell carcinoma (mRCC) [22]. Nevertheless, the response to immunotherapy varies among individuals, with some experiencing resistance or ineffectiveness. Consequently, identifying biomarkers is pivotal in refining treatment selection, reducing costs, and enhancing survival rates for mRCC patients. The expression of immune checkpoints within tumor cells and the tumor microenvironment (TME) significantly influences the response to immunotherapy [23,24]. Our investigation showed a positive correlation between EVI2A expression and numerous immune checkpoints, particularly PDCD1 and CTLA4. This correlation implies that individuals with elevated EVI2A expression may exhibit heightened sensitivity to immunotherapy. The findings from The Cancer Immunome Atlas (TCIA) align with this correlation, demonstrating that when at least one of CTLA-4 or PD-1 is positive, high expression of EVI2A is associated with increased sensitivity to immunotherapy.

Regarding the latter aspect, it is well-documented that the tumor microenvironment (TME) not only fosters tumor angiogenesis and metastasis but may also engender treatment resistance and failure [25]. Our exploration of the correlation between EVI2A and TME employed single-cell analysis, immune cell correlation analysis, and the ESTIMATE algorithm. Single-cell sequencing, an emerging biomedical technology, has gained prominence in studying the notable changes in tumor cell types and microenvironments observed across various solid tumors [26]. Intriguingly, our single-cell analysis study unveiled a specific upregulation of EVI2A in immune cells, particularly CD4+ T cells and CD8+ T cells. Additionally, immune cell correlation analysis consistently demonstrated a positive correlation between EVI2A and T cell follicular helper cells, CD4+ memory-activated T cells, and CD8+ T cells. Notably, CD8+ T cells were found to be significantly more prevalent in metastatic kidney cancer compared to primary kidney cancer (20.8% *vs*. 7.7% *vs*. 5.3%). An intrinsic characteristic of cancer is its ability to evade the immune response, with tumor cells mediating immune evasion by expressing PD-L1 ligands and interacting with PD-1 on T cells, thereby inhibiting T cell activity. Previous studies [27,28] have underscored the pivotal role of CD4+ T cells and CD8+ T cells in ineffective immunotherapy. These cell types play essential roles in the immune system, participating in antiviral and antitumor immune responses. CD4+ T cells, known as helper T cells, recognize viral infections and activate the immune system, while CD8+ T cells, referred to as cytotoxic T cells, identify and eliminate infected cells. The normal quantity and function of CD4+ T cells and CD8+ T cells are critical determinants of effective immune therapy. Inadequate levels of CD4+ T cells may compromise the immune system’s ability to combat viruses effectively, leading to infections. Similarly, insufficient CD8+ T cells may impede the immune system’s capacity to eliminate cancer cells, fostering cancer development. Hence, maintaining the normal quantity and function of CD4+ T cells and CD8+ T cells is paramount for effective immune therapy. In summary, EVI2A emerges as a novel biomarker with the potential to predict the response of Kidney Renal Clear Cell Carcinoma (KIRC) patients to immunotherapy.

Beyond its implications for immunotherapy, EVI2A expression also exhibits associations with targeted therapy. Our drug analysis results revealed that the IC50 values of sunitinib, pazopanib, and lapatinib were higher in patients with low EVI2A expression compared to those with high expression. This implies that patients with high EVI2A expression are more responsive to these drugs. Tyrosine kinase inhibitors (TKIs) are frequently employed as targeted therapeutic agents for the treatment of advanced or high-risk recurrent renal clear cell carcinoma. A mounting body of evidence indicates that the combination of targeted therapy and immunotherapy holds promise for patients with advanced renal cancer [29–31]. Our findings indicate that individuals with high expression of EVI2A exhibit heightened sensitivity to both immunotherapy and targeted therapy, suggesting that this subset of patients may derive substantial benefits from combination therapy. In summary, EVI2A holds the potential to emerge as a target in future treatments for renal clear cell carcinoma.

EVI2A is believed to be part of a cell-surface receptor complex, along with other proteins in the membrane. EVI2A has been linked to the development of tumors in mice and has been identified as an oncogene [32]. Furthermore, research by Qiu et al. [33] has revealed that EVI2A is a risk factor for oral tongue squamous cell carcinoma and plays a crucial role in the prognosis of this type of cancer. Despite extensive research, the specific function of EVI2A in the progression and outcome of KIRC is yet to be fully comprehended. We conducted a set of *in vitro* experiments using ACHN and 786-O cell lines to gain a deeper insight into its biological mechanism. Our results indicated that suppressing the expression of EVI2A impedes kidney cancer cells’ growth, spread, and mobility, aligning with our previous bioinformatics predictions. In our study, we discovered that high expression of EVI2A is associated with unfavorable overall survival (OS) and progression-free survival (PFS). Validation using GEO databases revealed a diagnostic receiver operating characteristic (ROC) greater than 0.9 for EVI2A. Additionally, we found a strong correlation between EVI2A and the TME and speculated that EVI2A might impact the efficacy of anti-tumor immunotherapy through various mechanisms. Our analysis of immune checkpoint and TCIA data revealed that patients with high EVI2A expression are more responsive to immunotherapy. These findings suggest that EVI2A may be a potential biomarker for prognosis, diagnosis, and immunotherapy in clear renal cell carcinoma. However, some things could still be improved in our study. First, due to the limited design of our research, the specific mechanism of action of EVI2A needs to be further explored. Secondly, our evaluation of the relationship between EVI2A and immunotherapy was conducted through a retrospective study, and additional prospective studies are necessary to affirm these findings.

## Conclusion

In summary, our comprehensive analysis, integrating bioinformatics scrutiny and *in vitro* experimentation, has discerned EVI2A as a prospective tumor biomarker for individuals with renal cell carcinoma. Furthermore, the discernible involvement of EVI2A in the immune microenvironment of renal cell carcinoma unveils novel perspectives that could significantly impact the landscape of immunotherapeutic approaches for this disease.

## Supplementary Materials

Fig S1. Univariate and multivariate Cox regression analysis of EVI2A

Fig S2. Co-expression analysis of EVI2A.

Fig S3. Quantitative analysis of EVI2A expression across the various cell groups.

Table S1. Data set information included in this study for EVI2A analysis

Table S2. Differential analysis was performed to obtain differentially expressed genes (DEGs) in EVI2A high and low groups

Table S3. GO enrichment analysis of EVI2A

Table S4. KEGG enrichment analysis of EVI2A

Table S5. Co-expression analysis of EVI2A

Table S6. Correlation between EVI2A expression and immune cells

Table S7. Correlation between EVI2A expression and immune checkpoints

## Data Availability

All data are from open access databases. TCGA database, GEO database and TCIA database.

## Abbreviations

KIRC: Kidney renal clear cell carcinoma
TCGA: The Cancer Genome Atlas
GEO: Gene Expression Omnibus
TCIA: The Cancer Immunome Atlas
TME: Tumor microenvironment
TISCH: Tumor Immune Single-cell Hub
PFS: Progression-free survival
OS: Overall survival
DEGs: Differentially expressed genes
GO: Gene Ontology
KEGG: Kyoto Encyclopedia of Genes and Genomes
IC50: Half-maximal inhibitory concentration values
TKIs: Tyrosine kinase inhibitors
mRCC: Metastatic renal cell carcinoma

## Supplementary Materials

The supplementary material is available online at https://doi.org/10.32604/or.2024.050851.
