# Supplementary figures and images for: Integrative bioinformatics and in vitro exploration of EVI2A expression: unraveling its immunological and prognostic implications in kidney renal clear cell carcinoma
Source: Oncol Res. 2024 Oct 16;32(11):1733–46. doi: 10.32604/or.2024.050851 (PMC11497181; doi:10.32604/or.2024.050851)

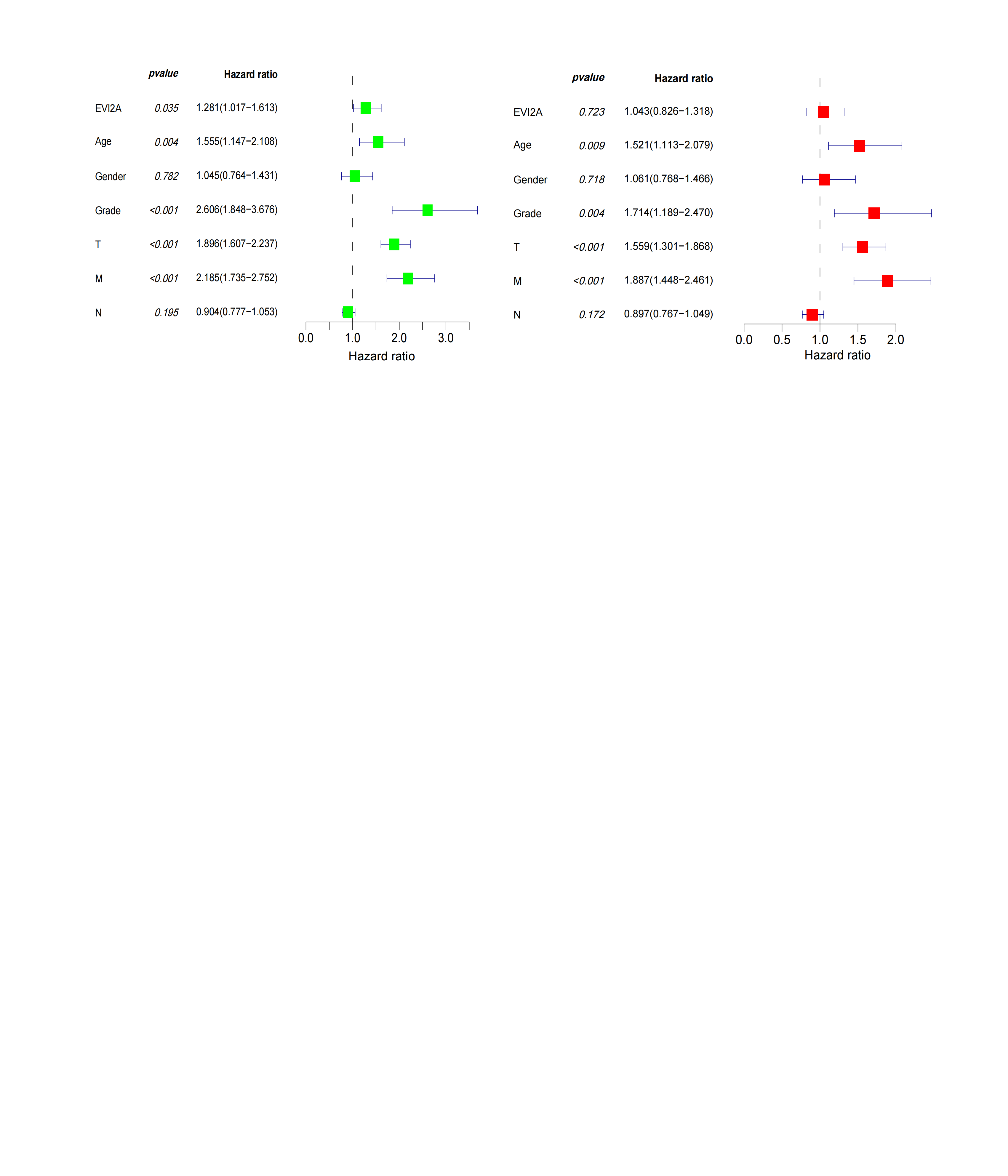

Supplement: Fig S1. [file OncolRes-32-50851-s001.tif]

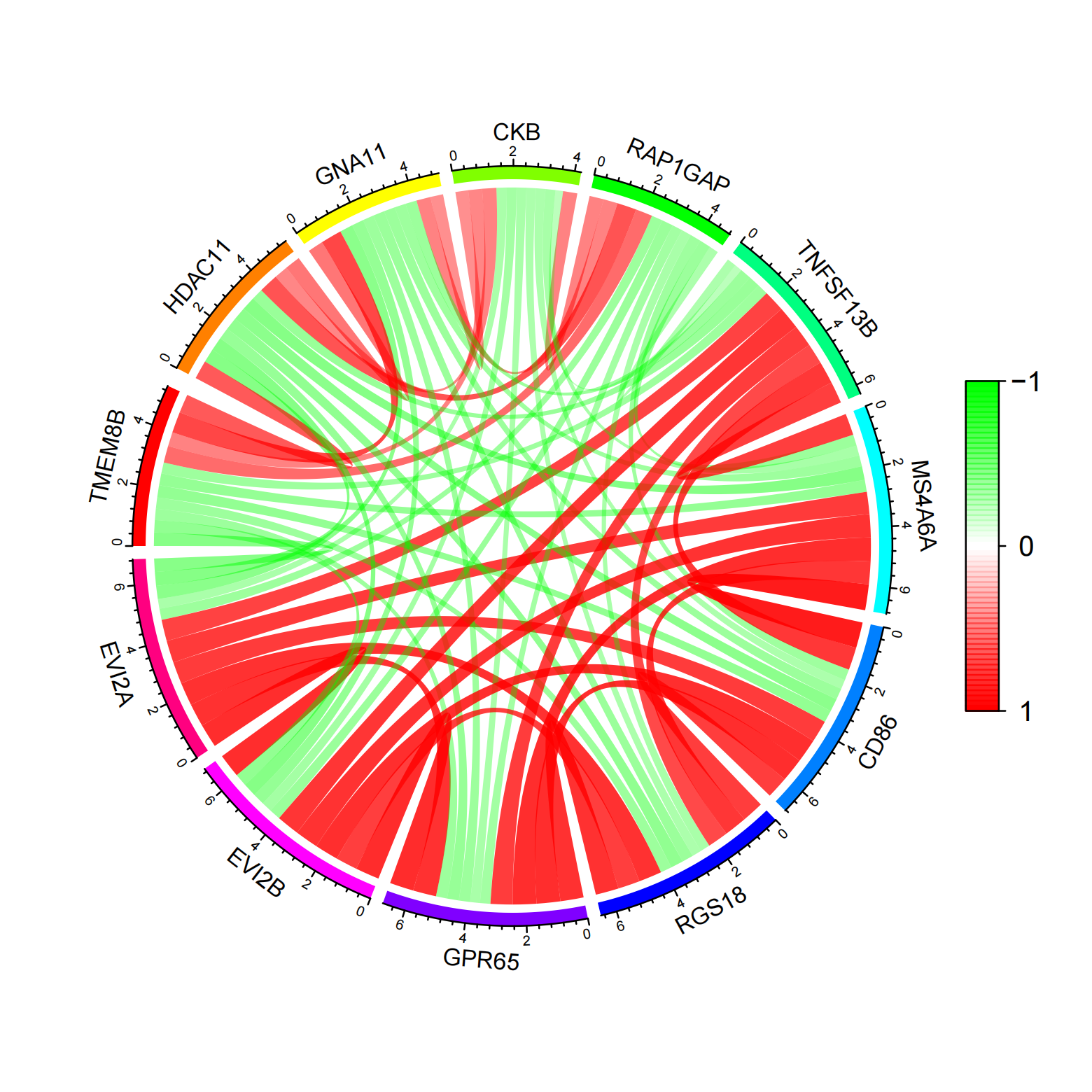

Supplement: Fig S2. [file OncolRes-32-50851-s002.tif]

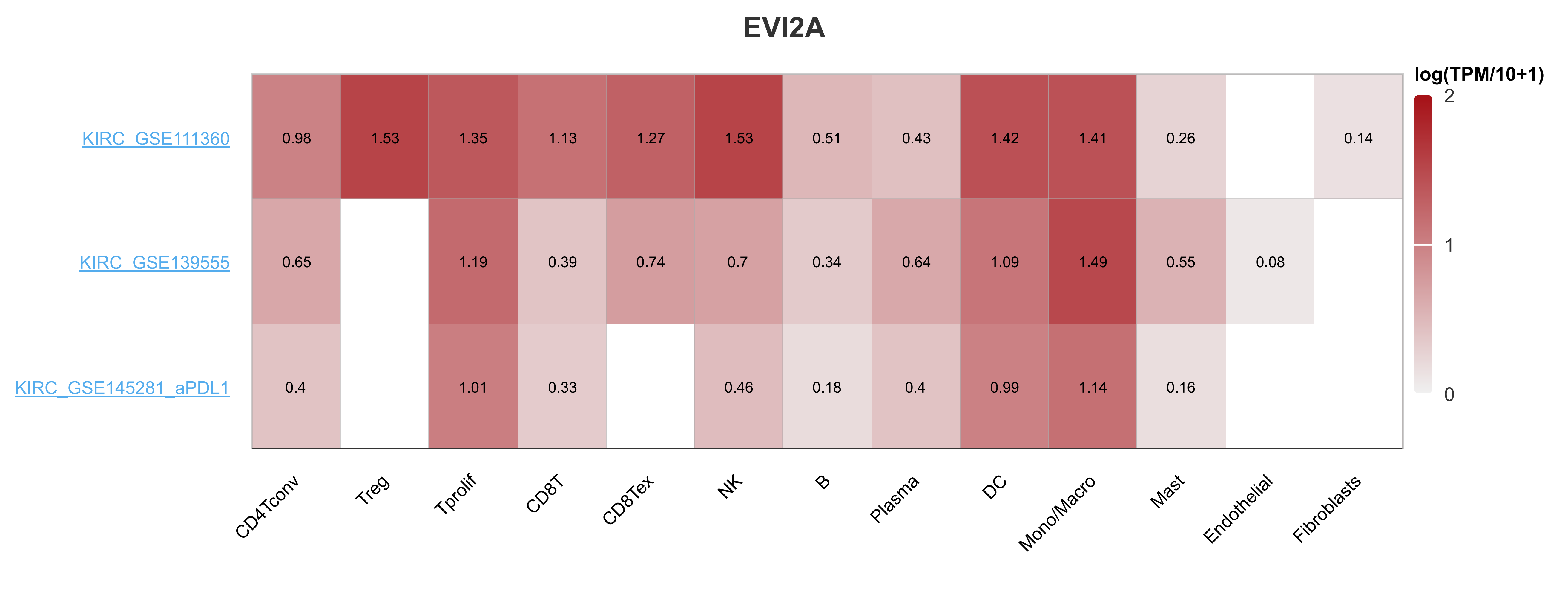

Supplement: Fig S3. [file OncolRes-32-50851-s003.tif]
