# Supplementary material for: Integrative bioinformatics and in vitro exploration of EVI2A expression: unraveling its immunological and prognostic implications in kidney renal clear cell carcinoma
Source: Oncol Res. 2024 Oct 16;32(11):1733–46. doi: 10.32604/or.2024.050851 (PMC11497181; doi:10.32604/or.2024.050851)
Supplement: Table S1. [file OncolRes-32-50851-s001.docx]

| **Table-S1: Data set information included in this study for EVI2A analysis** | | | | | |
| --- | --- | --- | --- | --- | --- |
| **Accession number** | **Number of KIRC patients** | **Number of normal patients** | **Number of tumor patients** | **Platform** | **Platform** |
| TCGA | 613 | 72 | 541 | TCGA | TCGA |
| GEO: GSE53000 | 62 | 6 | 56 | GPL6244 | Affymetrix Human Gene 1.0 ST Array |
| GEO: GSE53757 | 144 | 72 | 72 | GPL570 | Affymetrix Human Genome U133 Plus 2.0 Array |
| GEO: GSE66272 | 54 | 27 | 27 | GPL570 | Affymetrix Human Genome U133 Plus 2.0 Array |
| GEO: GSE68417 | 49 | 20 | 29 | GPL6244 | Affymetrix Human Gene 1.0 ST Array |
